# What next in measles control for Karnataka, India?

**DOI:** 10.1186/1753-6561-6-S1-P13

**Published:** 2012-01-16

**Authors:** MR Raveesha

**Affiliations:** 1 Institute of Public Health, Bangalore, India

## Introduction

Worldwide, measles kills 400 people every day, more than 90% of them being under five years of age. Three out of four measles deaths happen in India, the only country that has not yet introduced a second dose of measles vaccine. Prevention of deaths due to measles is key to achieve fourth millennium development goal of reducing under-five mortality by two thirds by 2015. India's progress in measles control is a major determining factor in global control of measles.

There is limited literature available on measles epidemiology in India. No measles surveillance was done before 2006. The measles surveillance programme, based on an existing flaccid paralysis surveillance system, was launched in four southern states of India in 2006, including Karnataka. In this paper I attempt to describe the epidemiology of measles in Karnataka and to identify ways to improve measles control in the state.

## Methods

I synthesised the spatio-temporal distribution of measles cases and outbreaks in Karnataka by collating weekly surveillance reports and outbreak investigation lists over four years (2006-2009). I reviewed the international literature on determinants of measles outbreaks. I specified the contextual demographic and socio-cultural determinants of measles outbreaks and vaccination coverage in the state of Karnataka through logistic regression multivariate analysis.

## Results

Measles surveillance data from 2006 to 2009 revealed that measles is endemic in Karnataka, with frequent outbreaks. The notification rate of measles is 10.94 cases per 100,000 populations per year. Seasonality of notified measles cases characteristically increases between November and April and decreases from May to October.

There were 163 confirmed outbreaks in Karnataka during the four years (2006-2009). Measles outbreaks were happening consistently in the northern part of the state and sporadically in the southern part. Every year, 22 out of 179 blocks, referred to as high-risk blocks (of which 21 in northern Karnataka) reported outbreaks. Of those affected by measles, 51% were under five years and 38% were in the age group of five to nine years. In the age group one to four years, only 46% of the measles affected had received a documented dose of measles vaccine.

The vaccine efficacy in the high-risk blocks was between 80% and 90%. The duration of these outbreaks from the date of appearance of rash in the first case to last case ranged from 7 days to 120 days with median of 39 (first quartile 15, third quartile 63) days. The total number of measles cases in each outbreak (across 163 outbreaks) ranged from 20 to 256 with a median of 38 (first quartile 25, third quartile 74) cases.

Through bivariate analysis, we found that block's probability to have an outbreak was positively associated with a number of variables including low vaccination coverage in the block; decadal population growth rate of the block; scheduled caste and scheduled tribe population; below poverty line population; agriculture as occupation; poor housing conditions; low living index; and low literacy level.

However multivariate analysis revealed that only the low vaccination coverage (below 84.9%, Odds ratio 9.8) and decadal population growth rate (more than 19.6%, Odds ratio 6.1) remained statistically significant as independent predictors of measles outbreaks in a block.

The surveillance programme in Karnataka complied with the standards of timely and complete reporting (both over 90%) since 2006. Out of 172 suspected outbreaks, 163 were serologically confirmed.

## Discussion

Refined understanding of measles epidemiology allows for appropriate action for measles control, which is critical to reduce under-five mortality. In Karnataka, both an organisational and a geographical focus are needed in measles control programme. Further reduction in measles incidence and outbreaks may be attained by adding a second dose to the routine vaccination scheme in the whole state and by conducting catch-up campaigns in northern Karnataka, where most high-risk blocks lie.

Alongside theses priority interventions, surveillance can be strengthened, and case management can be improved with more efficient vitamin A administration and inclusion of outbreak response immunisation.

According to the international literature, three major factors contribute to measles outbreaks: inadequate routine vaccination coverage, inadequate vaccine efficacy and situations in which there is an accumulation of unprotected population coming into direct contact with measles cases.

